# 

**Published:** 1935

**Authors:** 


### CONTENTS.

| | Page |
|---|---|
| Men-Midwives of the Past. By Miles H. Phillips, M.D., F.R.C.S., F.C.O.G. | 83 |
| Eczema and Dermatitis. By Norman Burgess, M.D., M.R.C.P. | 103 |
| Medical Treatment of Hyperthyroidism. By J. Innes Noble, M.B., Ch.B., F.R.C.S. | 113 |
| Reviews of Books | 125 |
| Editorial Notes | 130 |
| Meetings of Societies | 134 |
| The Medical Library of the University of Bristol | 135 |
| Publications Received | 139 |
| Local Medical Notes | 140 |

